# Bioprobes Based on Aptamer and Silica Fluorescent Nanoparticles for Bacteria *Salmonella typhimurium* Detection

**DOI:** 10.1186/s11671-016-1359-z

**Published:** 2016-03-16

**Authors:** Qiu-Yue Wang, Yan-Jun Kang

**Affiliations:** College of Laboratory Medicine, Hunan University of Medicine, Huaihua, Hunan 418000 China; Wuxi Medical School and Public Health Research Center, Jiangnan University, Wuxi, Jiangsu 214122 China

**Keywords:** Bacteria *Salmonella typhimurium*, Aptamer, Silica, Fluorescent nanoprobes

## Abstract

In this study, we have developed an efficient method based on single-stranded DNA (ssDNA) aptamers along with silica fluorescence nanoparticles for bacteria *Salmonella typhimurium* detection. Carboxyl-modified Tris(2,2′-bipyridyl)dichlororuthenium(II) hexahydrate (RuBPY)-doped silica nanoparticles (COOH-FSiNPs) were prepared using reverse microemulsion method, and the streptavidin was conjugated to the surface of the prepared COOH-FSiNPs. The bacteria *S. typhimurium* was incubated with a specific ssDNA biotin-labeled aptamer, and then the aptamer-bacteria conjugates were treated with the synthetic streptavidin-conjugated silica fluorescence nanoprobes (SA-FSiNPs). The results under fluorescence microscopy show that SA-FSiNPs can be applied effectively for the labeling of bacteria *S. typhimurium* with great photostable property. To further verify the specificity of SA-FSiNPs out of multiple bacterial conditions, variant concentrations of bacteria mixtures composed of bacteria *S. typhimurium*, *Escherichia coli*, and *Bacillus subtilis* were treated with SA-FSiNPs.

In addition, the feasibility of SA-FSiNPs for bacteria *S. typhimurium* detection in chicken samples was assessed. All the results display that the established aptamer-based nanoprobes exhibit the superiority for bacteria *S. typhimurium* detection, which is referentially significant for wider application prospects in pathogen detection.

**Electronic supplementary material:**

The online version of this article (doi:10.1186/s11671-016-1359-z) contains supplementary material, which is available to authorized users.

## Background

Bacteria *Salmonella typhimurium* (bacteria *S. typhimurium*) is a kind of food-borne zoonotic pathogen, which may cause food poisoning, acute gastroenteritis, and exogenous febrile disease and even lead to death [1, 2]. Especially, food poisoning caused by bacteria *S. typhimurium* is at the top of the bacterial food poisoning event list in the world [3]. Generally, bacteria *S. typhimurium* is mainly detected using morphological observation, immunological method, and molecular techniques nowadays [4–6]. Briefly, the morphological observation method only provides the shape, size, and dyeing property (gram positive or gram negative) of bacteria, which cannot provide the specific types of information of the bacteria the researchers look for. Although the immunological method, like enzyme-linked immunosorbent assay (ELISA), is a widely used method for bacteria detection, the procedure is complex and time-consuming. The PCR-based methods are popular at present, but they are easily contaminated and time-consuming sometimes. Therefore, to develop a fast and effective method for bacteria *S. typhimurium* detection to remedy the extant weaknesses is essential in a pathogen-monitoring program as well as clinic diagnosis.

In recent years, the aptamer has attracted tremendous interest because of its excellent features for pathogen detection. Aptamers are kinds of synthetic peptide or oligonucleotide molecules which can bind to the target molecule firmly. With advantage of high affinity and specificity, the aptamers are prominent for detecting all kinds of targets, such as tumor cells, bacteria, virus, proteins, some small molecules (ATP), and even metal ions [7–11]. Compared with antibodies, aptamers would be promising recognition elements for bioanalysis because of their high affinity and specificity with various kinds of targets, easy preservation, facile modification, and good stability.

For the application in bacteria *S. typhimurium* detection, several aptamer probes had been selected and characterized using whole-bacterium SELEX [12–14]. According to the structural analysis of a selected aptamer and measurement of affinity constant between the aptamer and bacteria *S. typhimurium*, the bacteria *S. typhimurium* aptamer is a 40-base single-stranded DNA with Kd value (6.33 ± 0.58 nM), which indicates the high affinity and specificity of aptamers [14]. In addition, the single-stranded oligodeoxynucleotide anti-*S. typhimurium* aptamer has a lower molecular weight compared to antibodies.

The fluorescent dyes are materials commonly used as an indicator in bioanalysis, while the deficiency of photobleaching severely hinders the range of their applications. With the development of nanotechnology in recent years, fluorescent nanomaterials play an increasingly important role in bioanalysis [15]. Many researches had revealed that the nanometer material shell surrounding the dye molecule could prevent the dye from photobleaching. In addition, a larger amount of dye molecules entrapped inside the silica matrix would play the role of a signal amplifier. Then, the fluorescent core-shell nanoparticles, like the dye-doped silica nanoparticles, have attracted great attention due to their other good characters with respect to photostability, surface-to-volume ratio, modification, cost, biocompatibility, and hydrophilicity [16–19]. For instance, targeted fluorescence nanoprobes are prepared by conjugating the desirable biomolecules (antibody or protein) to the surface of the dye-doped silica nanoparticles, which have been widely used for tumor cell recognition and separation, bacteria labeling, and DNA ultrasensitive assay [20–22].

Tris(2,2′-bipyridyl)dichlororuthenium(II) hexahydrate (RuBPY) is a kind of hydrophilic positively charged fluorescent dye, which shows the fluorescence emission wavelength (610 nm) with the excitation wavelength at 450 nm. As mentioned above, compared with the free RuBPY dye, the RuBPY-doped silica nanoparticles take advantage of resistance to photobleaching due to dye molecules with a positive charge entrapped stably inside the negatively charged silica matrix, which prevents the potential quenching substance approaching the dye molecules [23, 24].

In view of the extraordinary properties of aptamer and fluorescent nanomaterials for target labeling and signaling [25–28], we prepare novel bioprobes based on single-stranded DNA (ssDNA) aptamers and RuBPY-doped silica nanoparticles for bacteria *S. typhimurium* detection, of which the availability was accessed in a variety of conditions.

## Methods

### Materials

Triton X-100, tetraethyl orthosilicate (TEOS), (3-aminopropyl)triethoxysilane (APTES), 1-ethyl-3-(3-dimethylaminopropyl)carbodiimide hydrochloride (EDC), *N*-hydroxysulfosuccinimide sodium salt (Sulfo-NHS), RuBpy, and streptavidin were purchased from Sigma Chemical Co. (St. Louis, MO). Ammonium hydroxide (25–28 wt%), n-hexanol, cyclohexane, acetone, alcohol, *N*,*N*-dimethylformamide (DMF), and succinic anhydride were purchased from China Pharmaceutical Group Shanghai Chemical Reagent Co., Ltd. Bacteria *S. typhimurium*, *Escherichia coli* (*E. coli*) DH5ɑ, and *Bacillus subtilis* (*B. subtilis*) were obtained from China Center for Type Culture Collection. The rhodamine B isothiocyanate (RBITC)-labeled bacteria *S. typhimurium* aptamer was purchased from Beijing Biosynthesis Biotechnology Co., Ltd. The biotin-labeled bacteria *S. typhimurium* aptamer: 5′-biotin-(CH2)_6_-AGTAATGCCCGGTAGTTATTCAAAGATGAGTAGGAAAAGA-3′ and the biotin-labeled random sequence: 5′-biotin-(CH2)_6_-TGTCATGACCCGTAGGTAGTCTTAGAAGACTAGGCACGTT-3′ were synthesized at Shanghai Sangon Biological Engineering Technology & Services Co. (China).

### Main Instrumentation

The size and uniformity of synthesized silica NPs were measured by means of transmission electron microscopy (JEOL, JEM100CXII, Japan). Photoluminescence was measured using a fluorescence spectrophotometer (F96PRO, China). Fluorescence image results were observed under an inverted fluorescence microscope (Nikon ECLIPSE TE2000-U, Japan).

### Preparation and Characterization of Streptavidin-Conjugated RuBPY-Doped Silica Fluorescence Nanoprobes

Carboxyl-modified RuBPY-doped silica nanoparticles (COOH-FSiNPs) were synthesized by the method described by Arriagada et al., and the detailed procedure referenced to Cai et al. [29, 30]. In short, the RuBPY-doped silica NPs were prepared through the polymerization reaction of TEOS and NH_4_OH with RuBpy in the water-in-oil microemulsion, which was made of Triton X-100, cyclohexane, and water. Then, the RuBPY-doped silica was amine modified using TEOS and APTES. Afterwards, the generated floccule was deposited using EDC and Sulfo-NHS and washed with acetone after centrifugation. By washing and resuspending with DMF solution, the products were reacted with succinic anhydride under nitrogen gas for 24 h with continuous stirring. Then, the prepared COOH-FSiNPs were washed with water and resuspended in PBS for the next step.

Two milligrams of COOH-FSiNPs, 1 mg of EDC, and 2.5 mg of Sulfo-NHS were added to 1 mL of 0.1 M PBS buffer (pH 7.4). The mixture was then incubated for 15 min at room temperature with gentle shaking. After the reaction completed, 50 μL of streptavidin diluted in PBS at a concentration of 1 mg/mL was immediately added to the solution, following a 3-h incubation with gentle shaking at room temperature. The particles were washed with 0.1 M PBS (pH 7.4) and then resuspended in 1 mL of 0.05 % BSA for 1 h to block the free carboxylates. After the reaction completed, the prepared streptavidin-conjugated silica fluorescence nanoprobes (SA-FSiNPs) were washed with 0.1 M PBS buffer (pH 7.4) three times and then resuspended in 0.1 M PBS buffer (pH 7.4) for further use.

### Application of the Nanoprobes for Bacteria *S. typhimurium* Detection

One hundred microliters of 10 μM biotin-labeled anti-*S. typhimurium* aptamer and 1 mL of bacteria *S. typhimurium* diluted in PBS at a concentration of 80 cfu/mL were incubated for 60 min at 37 °C with gentle shaking. After the reaction completed, the suspension of bacteria *S. typhimurium* aptamer complex was centrifuged and washed with PBS three times and then resuspended in 1 mL of 0.1 M PBS (pH 7.4). One hundred microliters of SA-FSiNPs was immediately added to the suspension. After incubation for 60 min at 37 °C with gentle shaking, the mixtures were centrifuged (6000 rpm × 5 min) and washed with 0.1 M PBS (pH 7.4) three times to remove the free SA-FSiNPs. The probe-bacteria conjugates were finally resuspended in PBS and then smeared on a slide glass for fluorescence microscope observation. The corresponding control group was treated through the same procedure with exception of the anti-bacteria *S. typhimurium* aptamer which was replaced by a biotin-labeled random sequence aptamer.

### Streptavidin-Conjugated Silica Fluorescence Nanoprobe for Bacteria *S. typhimurium* Detection Under Multiple Bacterial Conditions

Three bacteria mixture groups were set in terms of the concentration gradient:

- Group A: 80 cfu/mL bacteria *S. typhimurium* + 40 cfu/mL *E. coli* DH5ɑ + 20 cfu/mL *B. subtilis*
- Group B: 40 cfu/mL bacteria *S. typhimurium* + 20 cfu/mL *E. coli* DH5ɑ + 80 cfu/mL *B. subtilis*
- Group C: 20 cfu/mL bacteria *S. typhimurium* + 80 cfu/mL *E. coli* DH5ɑ + 40 cfu/mL *B. subtilis*

The three groups were incubated with 300 μL of 10 μM biotin-labeled anti-*S. typhimurium* aptamer for 60 min at 37 °C with gentle shaking. After the reaction completed, three kinds of bacteria mixture were centrifuged and washed with PBS three times and then resuspended in 1 mL of 0.1 M PBS (pH 7.4). Two hundred microliters of SA-FSiNPs was immediately added to the bacteria mixture. Subsequent steps were as described in the “Application of the Nanoprobes for Bacteria *S. typhimurium* Detection” section.

### Fluorescence Nanoprobe for the Bacteria Detection in Chicken Samples

Five grams of minced chicken samples purchased from a supermarket and 100 μL of 1 × 10^3^ cfu/mL bacteria *S. typhimurium* were mixed in the triangular flask containing 50 mL of bacteria culture media (Mueller Hinton Broth). The mixture was then incubated for 12 h at 37 °C with gentle shaking. After the reaction completed, the culture media were centrifuged (1000 rpm × 5 min) and washed with PBS three times. The chicken samples were resuspended in 10 mL of 0.1 M PBS (pH 7.4). One hundred microliters of 10 μM biotin-labeled bacteria *S. typhimurium* aptamer was then added to the chicken samples dispersed in PBS. After incubation for 60 min at 37 °C with gentle shaking, the chicken sample suspension was centrifuged (5000 rpm × 5 min) with PBS three times. The chicken samples were then resuspended in 5 mL of 0.1 M PBS (pH 7.4). Two hundred microliters of SA-FSiNPs was subsequently added and then incubated for 2 h at 37 °C with gentle shaking. After centrifugation (1000 rpm × 5 min) and washing, the supernatant was transferred into a centrifuge tube and centrifuged (5000 rpm × 5 min). The probe-bacteria conjugates were finally resuspended in PBS and then smeared on a slide glass for fluorescence microscope observation. The procedures corresponding to the experimental groups were performed in the control groups except that the ground chicken samples were incubated with bacteria culture media in absence of bacteria *S. typhimurium*.

### Quantitative Analysis of Bacterial Cell Staining

Quantification of bacterial cell staining was performed using ImageJ software according to its standard manual.

## Results and Discussion

**Fig. 1:**
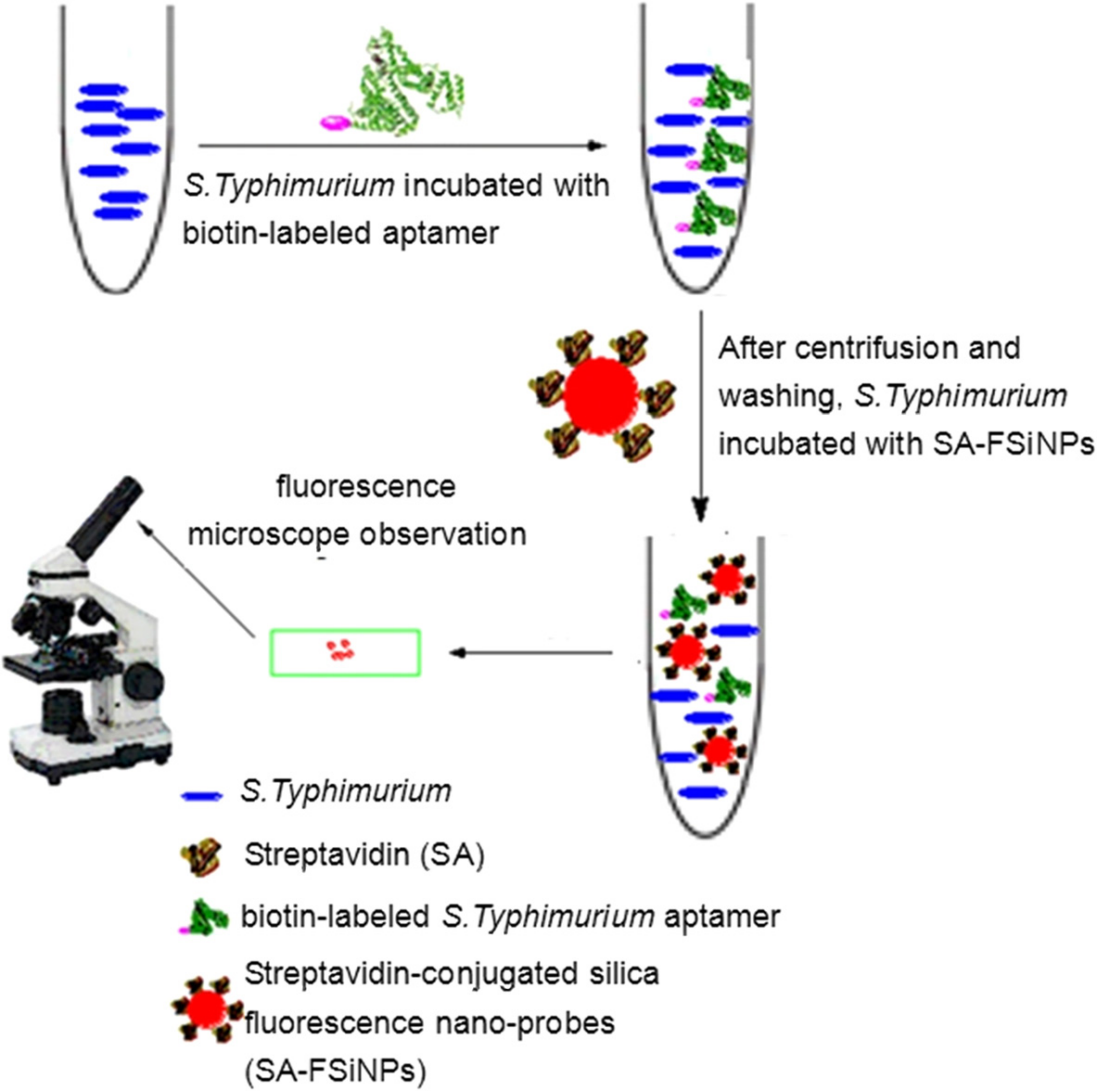
Overall schematic of fluorescent nanoprobes for bacteria *S. typhimurium* detection

In this research, we used the reported biotin-labeled ssDNA aptamer specific for bacteria *S. typhimurium* as the recognition elements, combining the silica fluorescence NP as the detection probe. The anti-*S. typhimurium* aptamer is a 40-base ssDNA, which shows high selectivity and affinity to the whole bacteria *S. typhimurium* [14]. Owing to the potential strong steric hindrance effects between the bacteria and the rigid NPs, the implementation for the probe would be impeded. Then in our study, the biotin-labeled aptamer was not directly conjugated to the SA-FSiNPs to avoid the conjugate (aptamer-biotin-SA-FSiNPs) formation. Instead, the bacteria *S. typhimurium* were firstly incubated with the free biotin-labeled aptamer that could result in mass molecules bounding to the bacteria surface, which would facilitate the subsequent labeling of SA-FSiNPs. So the bacteria *S. typhimurium* suspension was firstly incubated with the biotin-labeled aptamer. As expected, the biotin-labeled aptamers would bind to membrane proteins on the surface of the cell walls of the bacteria. After centrifugation and washing, the bacteria were then incubated with SA-FSiNPs. The fluorescence nanoprobes could bind to the surface of bacteria *S. typhimurium* because of the interaction between the biotin and the streptavidin. As for the control groups, the biotin-labeled random ssDNA could not specially bind to the membrane target bacteria, which results in no fluorescence signal under the microscope. Therefore, the bacteria *S. typhimurium* detection result can be recognized on the basis of striking fluorescence feature on the surface of detected bacteria. The overall schematic is described in Fig. 1.

### Characterization of Carboxyl-Modified RuBPY-Doped Silica Nanoparticles

**Fig. 2:**
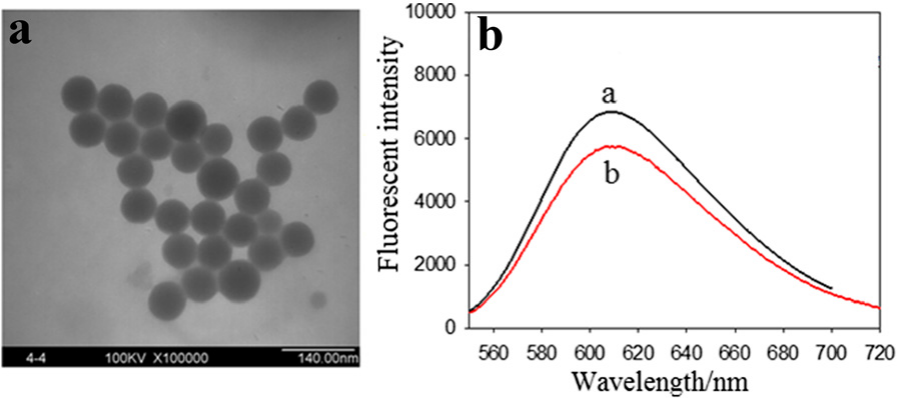
**a** TEM image of carboxyl-modified Rubpy-doped silica nanoparticles. **b** Fluorescence emission spectra of RuBPY dye (*curve a*) and carboxyl-modified Rubpy-doped silica nanoparticles (*curve b*)

The morphology information of synthesized silica NPs were measured by transmission electron microscopy. As shown in Fig. 2a, the NPs were uniform in shape with an average diameter of ~60 nm (the total number of NPs measured by the SIS image-processing software is 28, and the size of the measured NPs is 60 ± 5 nm). In addition, the fluorescence spectrum of COOH-FSiNPs was characterized by a fluorometer. As shown in Fig. 2b, compared with the fluorescence spectrum of free RuBPY dyes, COOH-FSiNPs have the same fluorescence emission wavelength (610 nm) with the excitation wavelength at 450 nm. Due to the mesoporous structure of silica, the RuBPY dyes entrapped inside the silica matrix are still excited [17, 31].

### Labeling and Imaging for Aptamer-Based Bacteria *S. typhimurium* Detection

**Fig. 3:**
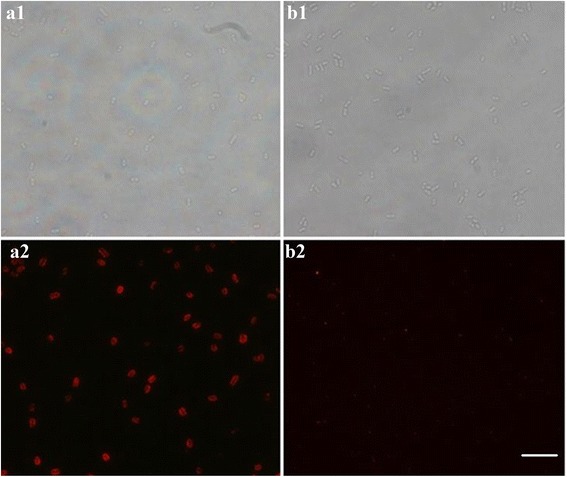
Fluorescence images of bacteria *S. typhimurium* labeled with the biotin-labeled aptamer and SA-FSiNPs (*A*) and biotin-labeled random single-stranded DNA and SA-FSiNPs (*B*). *a1* and *a2* represent the bright-field and fluorescence microscopy pictures in the experimental group, respectively. *b1* and *b2* represent the bright-field and fluorescence microscopy pictures in the control group, respectively. All images were obtained with fluorescence microscopy (100 × oil). *Scale bar* = 5 μm

To confirm whether the prepared SA-FSiNPs were practicable as a bacteria-labeling probe, bacteria *S. typhimurium* solution was firstly incubated with biotin-labeled anti-*S. typhimurium* aptamer. After centrifugation and washing, the bacteria were then treated with SA-FSiNPs. Unbound nanoprobes were washed away with PBS. The probe-bacteria conjugates were then imaged using fluorescence microscopy. The procedures corresponding to the experimental groups were performed in the control groups except that the biotin-labeled random sequence was used instead of the biotin-labeled bacteria *S. typhimurium* aptamer. As shown in Fig. 3, a1 and a2 represent the bright-field and fluorescence microscopy pictures in the experimental group, respectively. b1 and b2 represent the bright-field and fluorescence microscopy pictures in the control group, respectively. The fluorescence images were taken with the same contrast and brightness. Compared with the bright-field picture in the experimental group, bacterial colonies with significant red fluorescence were clearly observed in the fluorescence microscopy image, which indicates that a large number of SA-FSiNPs were bound to the surface of bacteria *S. typhimurium*. In contrast, the control group shows no red fluorescence of SA-FSiNPs under the fluorescence microscope, which indicates that there is no nonspecific binding between the bacteria *S. typhimurium* and nanoprobes. Then, we could agree that aptamers could recognize and bind to targeted bacteria with high affinity and specificity. In addition, fluorescent labeling of bacteria is attributed to the interaction between the streptavidin on the surface of nanomaterials and biotin on the surface of bacteria rather than the nonspecific binding between the nanomaterials and bacteria [32–34].

### The Specificity Verification of SA-FSiNPs

**Fig. 4:**
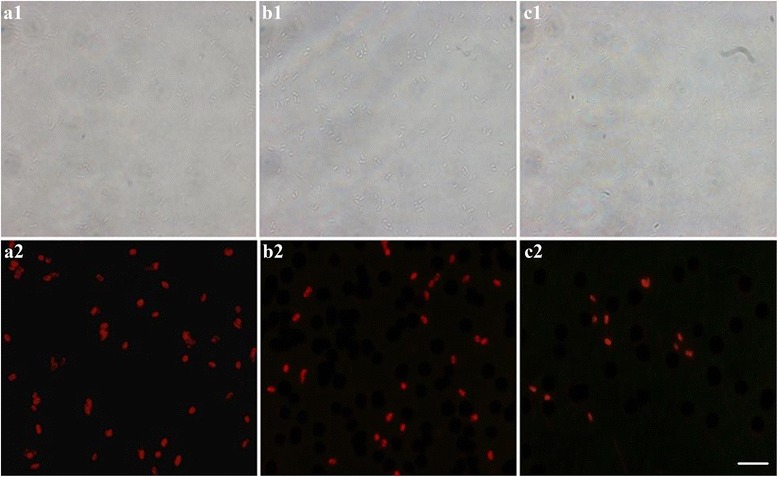
Fluorescence images of bacteria *S. typhimurium* in mixed bacteria samples. *a1*, *b1*, and *c1* represent the bright-field pictures. *a2*, *b2*, and *c2* represent the fluorescence microscopy pictures. *Scale bar* = 5 μm

To demonstrate the specificity of SA-FSiNPs for targeted bacteria detection, different concentrations of bacteria mixtures containing bacteria *S. typhimurium*, *E. coli*, and *B. subtilis* were treated with the same amount of nanoprobes. As shown in Fig. 4, a1 and a2 represent the bright-field and fluorescence microscopy pictures of group A bacteria, respectively. b1 and b2 represent the bright-field and fluorescence microscopy pictures of group B bacteria, respectively. c1 and c2 represent the bright-field and fluorescence microscopy pictures of group C bacteria, respectively. A dramatic decrease in the number of bacterial colonies with significant red fluorescence was observed as the bacteria *S. typhimurium* concentration in the bacteria mixtures decreased. In addition, to further demonstrate the NP probe binds specifically to bacteria *S. typhimurium* in mixed bacterial samples, pure bacteria *S. typhimurium* and pure *E. coli* DH5ɑ and pure *B. subtilis* were labeled with biotin-labeled bacteria *S. typhimurium* aptamers and SA-FSiNPs, respectively. Significant red fluorescence in bacterial colonies was observed in the fluorescence image of bacteria *S. typhimurium*, while no fluorescence was observed in the fluorescence images of *E. coli* DH5ɑ and *B. subtilis* (Additional file 1). The results show the great selectivity of nanoprobes for bacteria *S. typhimurium* labeling, which is due to the specific recognition between the aptamer and bacteria *S. typhimurium* and no nonspecific binding between the nanoprobes and bacteria.

### Photostability of Streptavidin-Conjugated Silica Fluorescence Nanoprobes

**Fig. 5:**
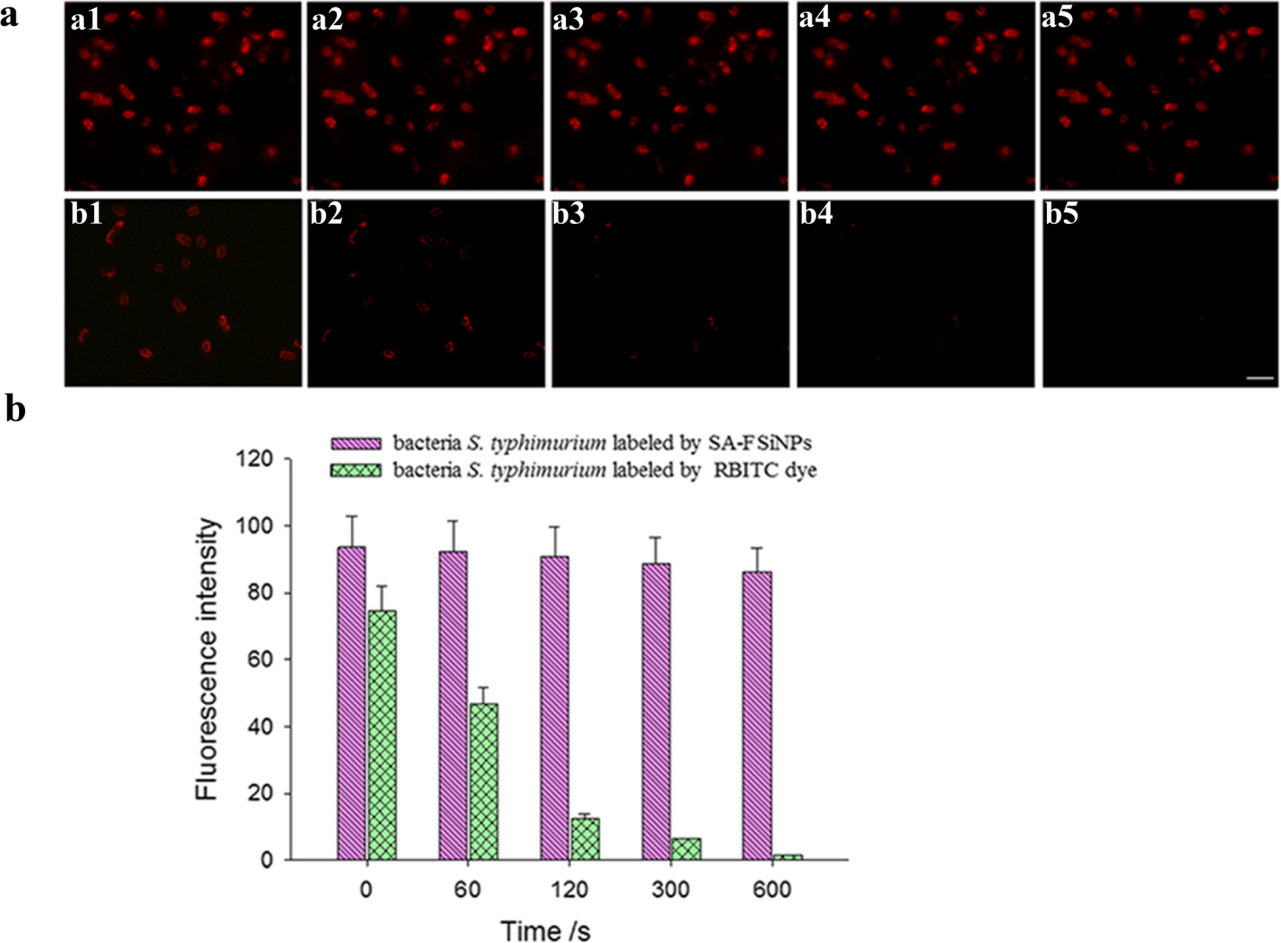
**a** Photostability comparison of SA-FSiNPs and RBITC dye. Bacteria *S. typhimurium* were labeled with the biotin-labeled aptamer and SA-FSiNPs (*a1*–*a5*) and RBITC-labeled aptamer (*b1*–*b5*), respectively, and excited for 10 min by the successive intense irradiation. The fluorescence images were acquired at 0 s (*a1* and *b1*), 60 s (*a2* and *b2*), 2 min (*a3* and *b3*), 5 min (*a4* and *b4*), and 10 min (*a5* and *b5*), respectively. *Scale bar* = 5 μm. **b** Quantitative analysis of bacterial cells, mean ± error bar (set at *P* < 0.05) versus different probes

It is known that the severe photobleaching of fluorescent dye molecules hinder its application for biological labeling. To verify the anti-photobleaching property of RuBPY dyes doped inside the silica matrix, bacteria *S. typhimurium* were labeled with SA-FSiNPs and commercially available RBITC-labeled aptamers and excited for 10 min by the successive intense irradiation simultaneously. The fluorescence images were captured at the time gradient of 0 s, 60 s, 2 min, 5 min, and 10 min. As shown in Fig. 5a, the prepared nanoprobes display a significant anti-photobleaching feature. The red signals were clearly distinguishable in the naked eyes even after successive intense irradiation for 10 min. The photostability of the encapsulated fluorescent dye molecules is prominent due to the protective effect of the silica shell, which can prevent from photodamaging oxidation [16, 35, 36]. Quantification of bacterial cell staining was performed using ImageJ software according to its standard manual and the reported method [37, 38]. All the bacterial cells in the images were selected individually using the freehand selection tool of the software. The mean pixel intensity of each bacterial cell on the fluorescence image was measured. Error bars reflect the standard error of the mean. Statistical significance was determined by Student’s *t* test. As shown in Fig. 5b, the fluorescence of RBITC was bleached quickly during 60-s irradiation.

### Application of the Bacteria *S. typhimurium* Detection out of Chicken Samples

**Fig. 6:**
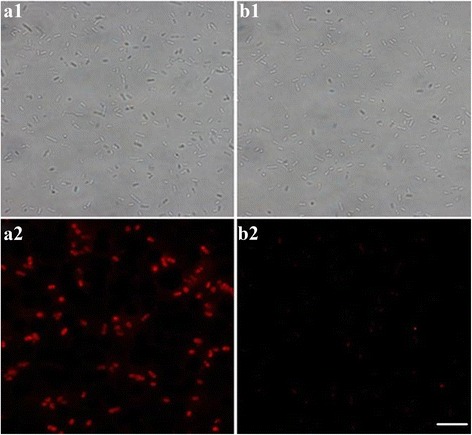
Fluorescence images of bacteria *S. typhimurium* treated with the biotin-labeled aptamer and SA-FSiNPs in the polluted chicken samples (*A*) and in the normal chicken samples (*B*). *a1* and *a2* represent the bright-field and fluorescence microscopy pictures in the experimental group, respectively. *b1* and *b2* represent the bright-field and fluorescence microscopy pictures in the control group, respectively. All images were obtained with fluorescence microscopy (100 × oil). *Scale bar* = 5 μm

To verify the feasibility of SA-FSiNPs in the practical application, the chicken samples contaminated with bacteria *S. typhimurium* were firstly detected using the established aptamer combining the SA-FSiNP treatment. The chicken samples without bacterial contamination process were set as the control groups. As shown in Fig. 6, a1 and a2 represent the bright-field and fluorescence microscopy results in the experimental group, respectively, while the b1 and b2 represent the information of the control group accordingly. A large number of bacterial colonies with significant red fluorescence were clearly distinguished under the fluorescence microscope in the experimental group, which indicates that the bacteria *S. typhimurium* present in the chicken samples are successfully labeled. In contrast, the control group shows no obvious fluorescence image, implying the control group samples are bacteria *S. typhimurium*-free. In addition, it is further confirmed that no nonspecific binding occurred between the nanoprobes and other potential bacteria in the chicken samples. So, these aptamer-based SA-FSiNPs are probably operative in the normal surveillance task, which is yet to be verified.

## Conclusions

A simple but effective method based on biotin-labeled aptamer and streptavidin-conjugated silica fluorescence nanoprobes for bacteria *S. typhimurium* detection had been established. The biotin-labeled anti-bacteria *S. typhimurium* aptamer shows high selectivity and affinity to membrane proteins of this food-borne pathogen. Because of the specific binding between streptavidin and biotin, the SA-FSiNPs can be effectively applied for the labeling of bacteria *S. typhimurium* in various complex backgrounds, which had been further confirmed in the multiple bacteria mixture and practical chicken samples. In addition, SA-FSiNPs display good photostability than the dye-labeled probes. Moreover, silica nanomaterials take advantage of good biocompatibility, which can serve as a necessary and useful supplement for the current detection methods.

## Additional file

Additional file 1: The results of bacterial detection tests in terms of higher concentrated samples. (DOC 864 kb)
