# Supplementary material for: Bioprobes Based on Aptamer and Silica Fluorescent Nanoparticles for Bacteria Salmonella typhimurium Detection
Source: Nanoscale Res Lett. 2016 Mar 16;11:150. doi: 10.1186/s11671-016-1359-z (PMC4794472; doi:10.1186/s11671-016-1359-z)
Supplement: Additional file 1: — The results of bacterial detection tests in terms of higher concentrated samples. (DOC 864 kb) [file 11671_2016_1359_MOESM1_ESM.doc]

**Supporting Information**

**Bioprobes based on aptamer and silica fluorescent nanoparticles for bacteria *Salmonella typhimurium* detection**

Qiu-Yue Wang^1^, Yan-Jun Kang^2*^

1 College of Laboratory Medicine, Hunan University of medicine, Huaihua, Hunan 418000, China

2 Wuxi Medical School& Public health research center, Jiangnan University, Wuxi, Jiangsu 214122, China

* Correspondence: [kangyj@jiangnan.edu.cn](mailto:kangyj@jiangnan.edu.cn)


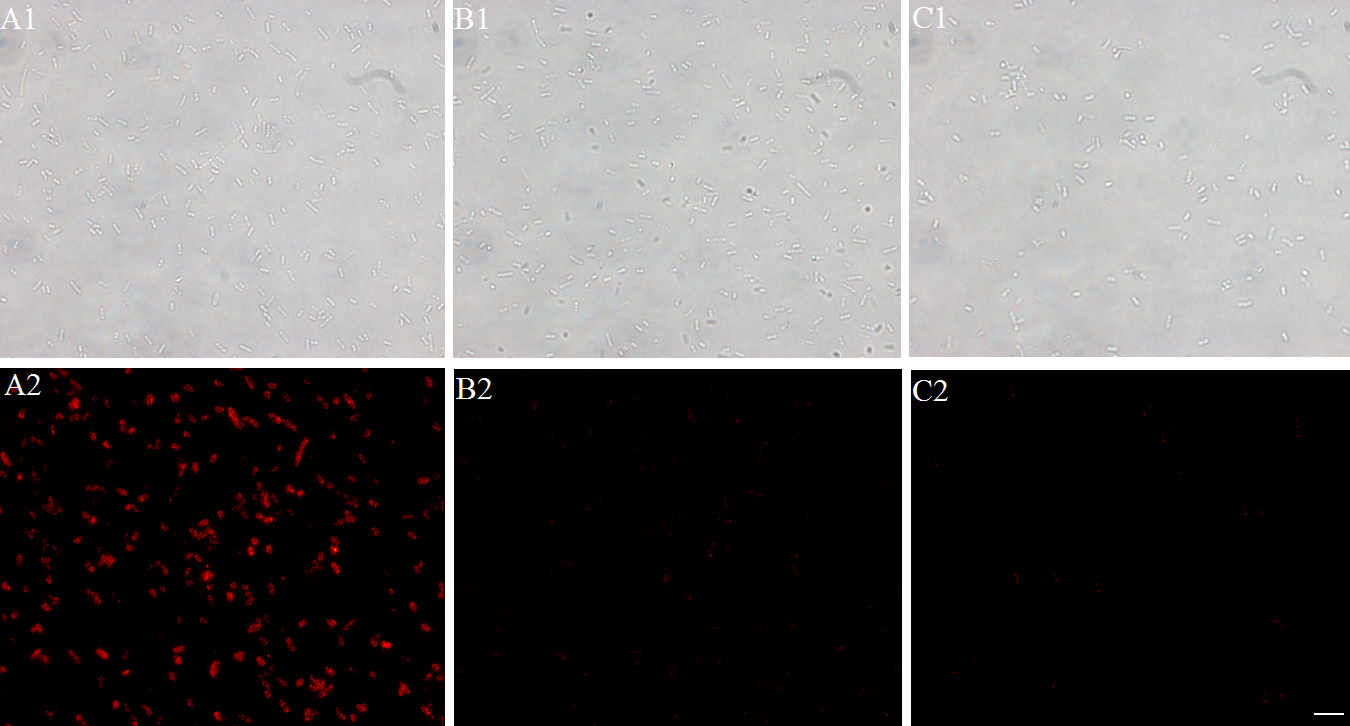


Figure S1(A)

Figure S1(B)

**Fig. S1.** (A) Fluorescence images of pure bacteria *S. typhimurium*, pure *E. coli* DH5ɑ, and pure *B. subtilis* were labeled with the biotin-labeled aptamer and SA-FSiNPs respectively. A1 and A2 represent the bright-field and fluorescent microscopy pictures of bacteria *S. typhimurium*, respectively. B1 and B2 represent the bright-field and fluorescent microscopy pictures of *E. coli* DH5ɑ, respectively. C1 and C2 represent the bright-field and fluorescent microscopy pictures of *B. subtilis*, respectively. All images were obtained with fluorescent microscopy (100×oil) and Scale bar = 5 μm. The bacteria concentration is 1.0×10^4^ cfu/mL. (B) Quantitative analysis of bacterial cells, mean ± error bar (set at P<0.05) versus different bacteria samples.
